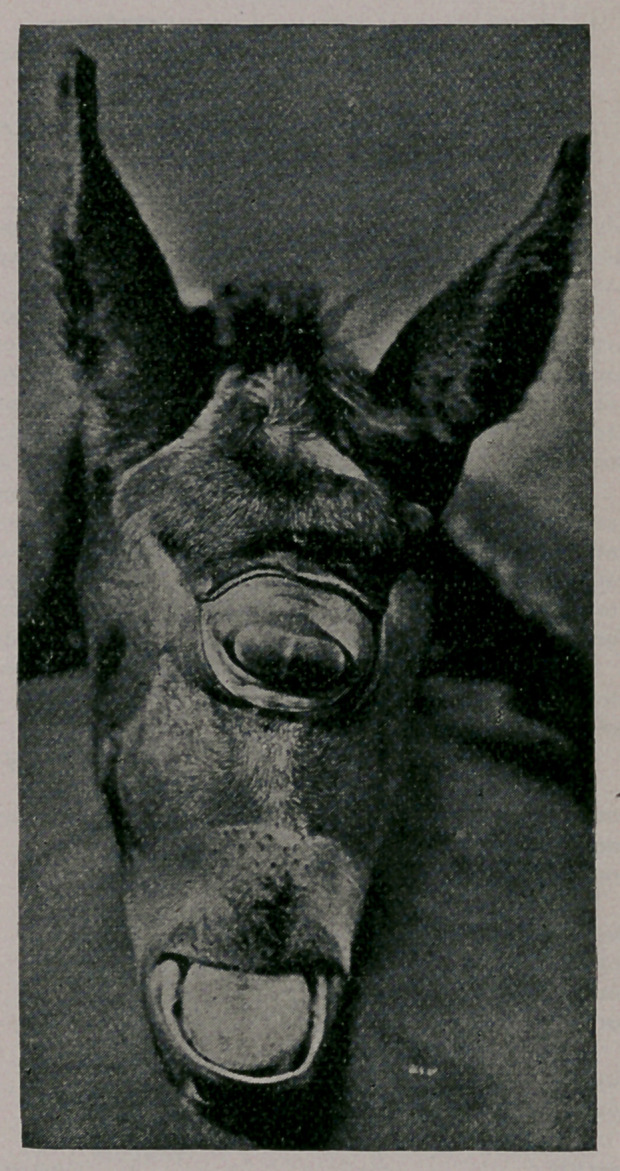# Case Department

**Published:** 1889-10

**Authors:** 


### CASE DEPARTMENT.

DIFFICULT PARTURITION SUCCEEDED BY PARTURIENT LAMINITIS.—Subject, a highly bred trotting mare, in foal to a son of Electioneer, aged 10, strong and vigorous, primapara. The foal presented with nose turned towards breast and fore feet in the vaginal passage. The rectum was everted, and the vulva enormously engorged. No portion of the fœtus protruded. Labor pains had ceased. Subject lying cold and motionless, and had been in this position about six hours. On opening the lips of the vulva, a large quantity of semi-coagulated venous blood escaped. Two large rents were found in the posterior walls of the vagina, one on each side, through which the feet extended through the surrounding tissues. The skin in neighborhood of perinæum, on each side of the vulva, prevented the feet from coming through. The feet were quickly slipped out of the pockets thus formed. This was accompanied by a gush of venous blood. The bloodvessels were plainly visible and very much engorged. Little difficulty was encountered in tipping the nose up into position and the foal, which was dead, was delivered, but not without considerable traction. It was very large, so much so, that I had it weighed. Weight 149 lbs. The mare was carefully looked after by giving moderate doses of stimulants, hand-rubbing, and clothing. After a couple of hours she got up, and rounded to quickly. The afterbirth was retained about ten hours. The mare was made as comfortable as possible, and not disturbed until next day. Appetite fairly good, pulse full and strong, 65°, temp. 104°. Disinclination to move about, except when forced. Did not think this important, as I thought the labor she had gone through was sufficient cause. There was a spasmodic contraction of the muscles of nigh hind limb, the vulva, enormously engorged, was spread apart, and large quantities of clotted blood removed. From the deep lacerations, which were plainly visible, with a strong light reflected on the vagina, found that the whole surface of canal was more or less lacerated and torn. The parts were dressed frequently with a solution of permanganate of potash, a superior dressing for wounds of this nature. The discharge was brown in color, streaked with pus of a clearer color, and shreds of membrane, very offensive. The next day parturient laminitis was evident. The usual treatment was adopted but without much avail ; the symptoms were modified, but on the twelfth day, although the animal was standing on sawdust, the moment one fore foot was picked up she would fall down. Descent of the sole was quite apparent, the mare was put in slings, and shod with leather soles, with manifest advantage. But from this, the subject "hung fire" so to speak, until about the sixth week, when quite a decided improvement was apparent, and has been gaining ground slowly. She is now in good spirits, and thriving, but her usefulness is very much impaired, not being fit for fast work, or even moderate driving, and very doubtful if it would be wise to breed her again. The inferior commissure of vulva is everted, but does not cause interference. The glutei muscles of nigh hind limb, the one in which occurred the spasmodic twitching, have become atrophied. I have had a large experience in matters relating to parturition, but cannot call to mind anything to be compared with this case in point of severity, and believe the termination, *i, e.*, pumice foot, exceptional.

It might be of interest for me to state that about an hour after this operation my arm from the shoulder to the wrist was covered with a vesicular eruption, which in two days became pustular. The arm was kept swathed with carbolized oil, by order of my physician. No discomfort ensued more than a burning, prickly sensation, with an occasional quick, sharp lancinating pain, but not severe enough to keep me from work. The process of desquamation of crusts and scales was not complete for about three weeks. I was obliged by force of circumstances to operate on a case of dystocia in the meantime, the arm well saturated with carbolized oil. No evil result followed.

DIARRHŒA IN FOALS.—Diarrhœa has been from some cause very prevalent this season throughout the Province. In fact almost epizootic. The only possible cause I can find, is the bad condition of oats. All through the country it is impossible to find a sound bushel of grain, it being affected with must. Oats with us are a staple food. Very little if any corn is consumed, but in some sections cornmeal is fed, yet only to a limited extent. White oats imported from Ontario are sound. Last year owing to the bad harvesting season, the bulk of this grain was not garnered until October, some in November and December, and some not taken from the field at all, and particularly so from localities near the coast.

Diabetes Insipidus has been exceedingly common, but quickly cured by a change to sound white oats. Whether this would have an effect on the intestinal tract of suckling foals I am unable to state, but the fact is noteworthy, where an entire change was made in the feed many foals recovered, many of the mares at the time being diabetic. As a medicinal adjuvant I would confidently recommend from 5 to 15 grains of lactopepsin given frequently, and if much pain is manifested by the little animal, yawning, bowing of the neck, stiffening of the limbs, with very loose discharges, 1/8 grain of hydrochlorate morphia and 1/2 drachm of ol. theobromo. made into a suppository will be found beneficial. It may be repeated in three or four hours if necessary. The sporific effect of this drug is quickly produced in foals, the excitant action frequently seen after hypodermic injections in aged horses being absent. At least this has been my experience, and have used it successfully in many cases. This scourge has been widespread, and its frequent fatal results have discouraged many breeders.

Post Partum—Paralysis—The subject, an aged carcarriage mare, had been subject to frequent attacks of tympanitis during pregnancy, but not of a violent nature ; parturition, was easy, and normal. Time of gestation ten months. Foal strong and healthy. The mare very uneasy after delivery, making frequent efforts to get up, but could not, the posterior extremities being in a state of paralysis. There was a continued rhythmic movement of the lower extremities. The escape of large volumes of flatus, together with distension of the abdominal walls, and much pain, at once indicated another attack of flatulent colic. The animal soon commenced to throw itself violently about, continuing in this manner for nearly ten hours, before the symptoms were successfully overcome, the owner persisting that she should be "slung." This operation was tried twice against my wishes and advice, and had to be abandoned, as the animal had no use of its posterior extremities. After the tympanitis had disappeared, she laid quietly for an hour, and without the slightest warning or effort got quietly on her feet, much to the surprise and delight of the owner.

JAMES H. FRINK, V.S.,
*Government Veterinary Inspector.*
*St. John, New Brunswick.*

STRANGE FREAK OF NATURE.—On the morning of the 20th of June, I was called to attend a difficult case of parturition in a mare. Upon examination I found an anterior presentation with a deviation of the head upwards and backwards and the foal already dead. After a few unsuccessful attempts to bring the head into proper position, I decided upon embryotomy to save the mare. After a long and tedious operation the head and neck were severed from the body close to the shoulders, and each removed separately, and the accompanying cut represents the head. The body was that of a well formed colt, but the head had the superior maxilla two inches shorter than the inferior, and the premaxilla very rudimentary and only cartilaginous. The nasal chambers and posterior nares entirely absent, but a well formed pharynx and fully developed velum pendulum palati, so that had it been born alive existence would have been impossible. The eyes instead of being in their proper places were combined in one large eye located in the centre of the forehead. There were two pupils, two distinct lower lids and one upper, and the eye ball very prominent. As the mare has been kept near the railroad, it is believed by some that this strange freak of nature was caused by sudden fright at the train after night as the eye somewhat resembles the head-light of a locomotive.

S. C. ORR, V.S.,

*Manhattan, Kansas.*

## Figures and Tables

**Figure f1:**